# HIV-1 infection is associated with changes in nuclear receptor transcriptome, pro-inflammatory and lipid profile of monocytes

**DOI:** 10.1186/1471-2334-12-274

**Published:** 2012-10-29

**Authors:** Barbara Renga, Daniela Francisci, Claudio D’Amore, Elisabetta Schiaroli, Adriana Carino, Franco Baldelli, Stefano Fiorucci

**Affiliations:** 1 Department of Experimental and Clinical Medicine, University of Perugia, Perugia, 06100, Italy; 2 Department of Experimental Medicine and Biochemical Sciences, University of Perugia, Perugia, 06100, Italy

**Keywords:** Adhesion molecules, Chemokines, HIV infection, Nuclear receptors

## Abstract

**Background:**

Persistent residual immune activation and lipid dysmetabolism are characteristics of HIV positive patients receiving an highly active antiretroviral therapy (HAART). Nuclear Receptors are transcription factors involved in the regulation of immune and metabolic functions through the modulation of gene transcription. The objective of the present study was to investigate for the relative abundance of members of the nuclear receptor family in monocytic cells isolated from HIV positive patients treated or not treated with HAART.

**Methods:**

Monocytes isolated from peripheral blood mononuclear cells (PBMC) were used for analysis of the relative mRNA expressions of FXR, PXR, LXR, VDR, RARα, RXR, PPARα, PPARβ, PPARγ and GR by Real-Time polymerase chain reaction (PCR). The expression of a selected subset of inflammatory and metabolic genes MCP-1, ICAM-1, CD36 and ABCA1 was also measured.

**Results:**

Monocytes isolated from HIV infected patients expressed an altered pattern of nuclear receptors characterized by a profound reduction in the expressions of FXR, PXR, PPARα, GR, RARα and RXR. Of interest, the deregulated expression of nuclear receptors was not restored under HAART and was linked to an altered expression of genes which supports both an immune activation and altered lipid metabolism in monocytes.

**Conclusions:**

Altered expression of genes mediating reciprocal regulation of lipid metabolism and immune function in monocytes occurs in HIV. The present findings provide a mechanistic explanation for immune activation and lipid dysmetabolism occurring in HIV infected patients and could lead to the identification of novel potential therapeutic targets.

## Background

Despite improvements in antiretroviral therapy, to date, the life expectancy of HIV infected patients remains shorter than of the general population [1]. The reduction in the AIDS defining clinical conditions is in part compensated by the development of other co-morbidities, often connected to short or long term metabolic toxicities, as well as to an immune activation status, which can persist despite an effective therapeutic control of HIV replication [2]. A systemic immune activation drives to secretion of pro-inflammatory cytokines [3] and inflammatory markers are strong and independent predictors of mortality in HIV positive patients [4].

Lipid and cholesterol abnormalities are a common finding in HIV infected persons. Both the HIV infection, *per se*, and HAART have been linked to these metabolic abnormalities and both represent a well identified risk for the development of atherosclerotic complications [5].

Genes involved in lipid and cholesterol metabolisms in humans are governed by a family of ligand activated transcription factors [6].

Members of the nuclear receptor superfamily exert their regulatory activity integrating regulatory effects exerted by nutrients with intermediate metabolism by positively and negatively regulating the expression of effector genes [6]. One peculiar characteristic of some of these receptors is their ability to regulate the innate and adaptive immune system. This regulation is, in most cases, inhibitory in nature and results in a profound counter-regulation of inflammatory signaling in macrophages [7].

Nuclear receptors are important therapeutic targets [8], and their patterns of expression in HIV infection are unknown. We have recently demonstrated that the HIV matrix protein p17 regulates the expression of proinflammatory and proatherogenic genes as well as nuclear receptors FXR and PPARγ on monocytic cells in a STAT-1 dependent manner [9]. Noteworthy, we provided evidence that exposure of monocytes to nuclear receptors agonists abrogates the effect of p17 [9]. Supporting the notion that nuclear receptors ligands could be used in conjuction with conventional antiviral therapies in the treatment of HIV infection it has been recently demonstrated that the activation of nuclear receptor signaling contributes to the repression of HIV-1 replication in macrophages [10].

In the present study we analyzed the expression of various nuclear receptors in monocytic cells of HIV infected patients and correlated the expression of the nuclear receptor PPARγ with that of CD-36, a membrane transporter that mediates the lipid up-take on these cells.

## Methods

### Patients

We performed our pilot study on 24 subjects: 8 HAART- naïve HIV-infected patients, 8 HAART-treated HIV-infected patients with a viral load < 50 copies/ml from at least 6 months and 8 healthy controls. From February to May 2011 8 never-treated HIV infected patients meeting the clinical criteria of being ≥ 18 y old, without other infections (acute or chronic) and/or histories of dysmetabolic diseases (diabetes, dyslipidemias) were enrolled. In addition, we enrolled 8 HIV positive treated patients and 8 healthy controls, chosen among staff members, matched for age and BMI and satisfying the same exclusion criteria were enrolled. Authorization for collecting and using blood samples from these persons for *ex vivo* testing was granted by the ethical committee of Regione Umbria (Italy) on July 22, 2010 (authorization number CEAS 1654/20). An informed written consent was obtained from each participant to the study.

### Isolation of CD14-derived peripheral blood mononuclear cells (PBMC)

Peripheral whole blood samples (_~_ 30 ml) from patients and healthy controls were withdrawn in vacutainer tubes containing EDTA. PBMC were first isolated by density gradient centrifugation using the Hystopaque reagent (Pharmacia Biotech) and then positively selected using CD14 magnetic beads and LS columns according to the manufacturer’s instructions (Miltenyi Biotec). After isolation monocytes were lysated with 1 ml TRIzol reagent (Invitrogen).

### RNA extraction and real-time PCR

Total RNA was isolated from CD14-monocytes using the TRIzol reagent (Invitrogen) and reverse-transcribed using random hexamer primers and Super Script-II reverse transcriptase (Invitrogen). mRNA was quantified by Real-Time quantitative PCR on iCycler apparatus (Biorad) using specific primers: 18S: cggctaccacatccaaggaa and gctggaattaccgcggct; FXR: tacatgcgaagaaagtgtcaaga and actgtcttcattcacggtctgat; PPARα: acgattcgactcaagctggt and gttgtgtgacatcccgacag; PPARβ: gctgagaagaggaagctggt and cgatgtcgtggatcacaaag; PPARγ: gctggcctccttgatgaata and ttgggctccataaagtcacc; LXR: cgcactacatctgccacagt and tcaggcggatctgttcttct; PXR: agctggaaccatgctgactt and cacatacacggcagatttgg; VDR: gcccaccataagacctacga and agaagctgggagtgtgtctg; GR: ggcaataccaggtttcagga and tatgatctccaccccagagc; RARα: aggacaccatgaccttctcg and gtctccgcatcatccatctc; RXR: cctttctcggtcatcagctc and tgacggggttcataggtgag; MCP1: ccccagtcacctgctgttat and tcctgaacccacttctgctt; ICAM1: agcttctcctgctctgcaac and cattggagtctgctgggaat; ABCA1: gcttgggaagatttatgacagg and aggggatgattgaaagcagtaa; CD36: tttctgtatgcaagtcctgat and attaagccaaagaataggcac. PCR amplifications and data analysis were performed as described [11].

### Biochemical analysis

Serum levels of total cholesterol, tryglicerides and High density lipoproteins (HDL) were measured with enzymatic colorimetric methods (Cobas Integra 800, Roche, Germany).

### Quantification of HIV-1-RNA copy numbers in plasma

Plasma viral load was determined by quantitative reverse PCR using Cobas Amplicor HIV-1 Monitor Test, version 1.5, Ultrasensitive (Roche Diagnostic, Indianapolis, Indiana, USA). The limit of detection was 40 copies/ml plasma.

### Quantification of the CD4+ lymphocytes subset

CD4+ cell count was carried out in peripheral whole blood collected in EDTA by flow cytometry (CYTOMICS FC 500 BECKMAN COULTER). The absolute count was performed by Flow-count Fluorospheres on EPICS XL BECKMAN COULTER.

### Statistical analysis

All values are expressed as the mean ± SEM of n observations per group. Comparisons of more than 2 groups were made with a one-way analysis of variance with post-hoc Tukey’s tests. Correlation statistics between the mRNA levels of CD36 and PPARγ were performed using linear regression test. A P value of less than 0.05 was considered as statistically significant.

## Results

### Patient characteristics

The most relevant characteristics of the patients enrolled in this study are shown in Table 1. HAART-naïve HIV-infected patients had a baseline CD4+ cell count of 396.6 ± 121 cells/ml and detectable HIV RNA (50% > 10^5^ copies/ml). Under HAART therapy, the viral load was always < 50 copies/ml and the CD4+ cell count was significantly higher (851.1 ± 78). The analysis of the lipid pattern revealed that HIV infected patients had a robust reduction of total cholesterol and HDL levels, while triglyceride levels were slightly higher, in comparison to healthy donors. Under HAART, the levels of HDL and total cholesterol were similar to those of healthy donors, while the levels of triglycerides were not significantly higher; despite this, an upward trend was observed. No patients were under lipid-lowering medications. Overall, the metabolic findings were consistent with previously published data [5].

**Table 1:** Clinical characteristics of study participants

**Subjects**	**HEALTY(n=8)**	**HIV POSITIVE(n=8)**	**HAART(n=8)**	***P***
**Sex** (Male/Female)	4/4	6/2	6/2	-
**Age** (years)	45.2 ± 4.5	47.1 ± 4.6	44.8 ± 4.4	-
**BMI**	24.15 ± 1.29	23.35 ± 0.99	23.37 ± 0.65	-
**White Blood Cells** (cells/mm^3^)	6347 ± 685	4756 ± 1214	5740 ± 567	-
**CD4+** (cells/ml)	N/A	396.6 ± 121	851.1 ± 78 ^#^	# < 0.05
**Cholesterol** (mg/dl)	201.3 ± 7.7	133.5 ± 13.8 *	226.3 ± 13.4 ^#^	* < 0.05# < 0.05
**HDL** (mg/dl)	59.1 ± 5.9	28 ± 3.8 *	45.5 ± 5.9 ^#^	* < 0.05# < 0.05
**Triglycerides** (mg/dl)	86.1 ± 11.6	169.7 ± 50.1	181.6 ± 29	-
**Viral Load** (copies/ml)	N/A	840000±552000	< 50 ^#^	* < 0.05# < 0.05
**Therapy**	none	none	PI (n=5) and NNRTI (n=3)	-
**Blood pressure medications**	2	3	2	-

*P<0.05 versus healthy donors, #P<0.05 versus HIV patients.

PI, protease inhibitor; NNRT, non-nucleoside reverse transcriptase inhibitor.

N/A, not applicable.

### Analysis of nuclear receptor transcriptome

**Figure 1:**
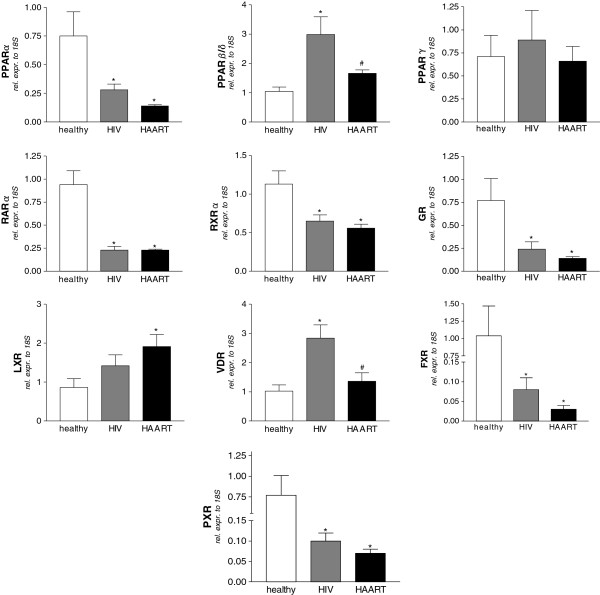
**Panels A-L. Nuclear receptor expression profile in CD14 monocytes isolated from healthy donors, HIV-infected and HAART-treated HIV-infected patients.** All values were normalized to relative expression of 18S mRNA and are expressed relatively to the expression of each gene in healthy donors. Analysis was carried out in triplicate (n=8 subjects / group). *P<0.05 versus healthy donors. #P<0.05 versus HIV-infected patients by ANOVA.

To evaluate the hypothesis that HIV infection may alter the expression of nuclear receptors in monocytes, we performed a semi-quantitative Real-Time PCR in cells isolated from the subjects reported in Table 1. As shown in Figure 1, we observed a strong down-regulation of mRNA levels for FXR, PXR, PPARα, GR, RARα and RXR both in HAART- naïve and in HAART-treated patients. In contrast, up-regulations of VDR and PPARβ mRNA levels were seen in HIV positive naïve patients and were almost completely restored by HAART. Noteworthy, LXR mRNA levels in HAART treated patients were significantly induced compared to healthy donors but not changed compared to HIV-infected patients. Finally, the analysis of the PPARγ gene demonstrated that the expression of this nuclear receptor was unmodified among the examined subjects. These observations lead us to conclude that HIV infection is associated with transcriptome alterations for many nuclear receptors and that HAART is only partially effective in counter-acting these.

### Analysis of lipid and inflammatory markers

**Figure 2:**
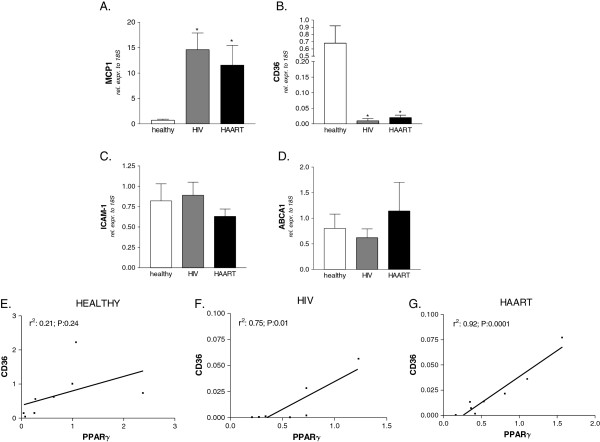
**Panels A-D. Relative mRNA expression of MCP-1 (A), CD36 (B), ICAM-1 (C) and ABCA1 (D) in CD14 monocytes isolated from healthy donors, HIV-infected and HAART-treated HIV-infected patients.** All values were normalized to relative expression of 18S mRNA and are expressed relatively to the expression of each gene in healthy donors. Analysis was carried out in triplicate (n=8 subjects / group). *P<0.05 versus healthy donors. #P<0.05 versus HIV-infected patients by ANOVA. Panels **E**-**G**. HIV positive patients but not healthy donors showed a significant correlation between mRNA levels of CD36 and PPARγ. R, correlation coefficient; P, P value.

Since nuclear receptors exert their functions at the interface between inflammation and lipid metabolism, we evaluated the expression of genes involved in immune regulation and lipid metabolism on monocytes isolated from both HAART- naïve and HAART-treated HIV-infected patients. As expected, a robust induction in the expression of the pro-inflammatory chemokine MCP-1 was observed in monocytes of HIV-infected patients, while the HAART mediated suppression of viral replication failed to normalize the mRNA level of this chemokine (Figure 2A). CD36 is a scavenger receptor mediating the uptake of oxidized low-density lipoproteins by monocytes. Here, we found that the expression of CD36 was strongly down-regulated in both HIV infected classes (Figure 2B). We also analyzed the relative mRNA expression of the cell adhesion molecule ICAM-1 as well as of the protein transporter ABCA1 involved in cholesterol efflux from monocytes and found that their expressions were unchanged in untreated HIV infection as well as in HAART (Figure 2C and D).

Noteworthy, a significant correlation between the mRNA levels of PPARγ and CD36 in HIV-infected naïve patients as well as in patients receiving HAART (Figure 2F and 2G) but not in healthy subjects was found (Figure 2E).

## Discussion

This study reports that HIV infection is associated with a profound dysregulation in the expression of genes encoding for members of the nuclear receptor super family in circulating monocytes.

It is well known HIV infection is associated to a chronic inflammatory condition and lipid abnormalities, with the latter being a consequence or concurrent cause of the inflammation state. Moreover, in treated patients, in spite of an effective control of the viral replication as well as in absence of pharmaco-induced dyslipidemias, a residual persistent chronic inflammatory status, which can limit the long-term effectiveness of therapy, can be detected [12].

We now provide evidence that, during the course of HIV infection, the expression of a large group of nuclear receptors is dramatically reset in monocytes, that this resetting is associated to a robust activation of circulating monocytes as demonstrated by 10–20 fold increase in the monocytic expression of MCP-1- RNA, and that HAART is not able to revert all the observed alterations. Specifically, we have obtained evidence that HIV infection causes profound down-regulations for FXR, PXR, PPARα, GR, RARα and RXRα. These down regulations were also observed under effective HAART.

A common motif of these transcription factors is their ability to activate counter-regulatory signals essential to reduce inflammation [7,8]. Indeed, besides the canonical anti-inflammatory activity of GR, FXR, PXR, PPARα, RARα and RXRα attenuate the inflammatory responses and their activations reduce immune dysfunction-driven inflammation in a variety of experimental and clinical settings [7,13]. Furthermore, data from a variety of gene deficient mice strongly supports the general notion that ablation of these receptors leads to the development of a deregulated immunity and causes metabolic dysfunction [7,13]. The present study is, therefore, a general model to examine the effect of viral infection and to further understand how HIV virus-induced inflammation and immune-dysfunction progress in human disease.

The detected alterations in the nuclear receptor expression are probably an indirect effect of viral replication, considering that only a very low number of circulating monocytes is infected by HIV [14]. These changes could be determined by either the chronic inflammatory process, as seen in atherosclerosis, type 2 diabetes mellitus, intestinal chronic inflammation, including inflammation driven gastrointestinal cancers, or the residual immune activation and microbial translocation associated with increased levels of LPS [15-20]. This could also arise from the action of some HIV proteins that can persist for a long time- in the body of virologically controlled patients [21]. This latter hypothesis could explain the persistent alterations observed in the group of HAART-treated HIV-positive subjects. Indeed, recently we have documented that the p17 HIV protein, which can persist in lymph nodes germinal centers of patients effectively treated [21], was able to down regulate PPARγ and FXR expressions in both THP1 cells and in healthy donors monocytes. Moreover, the serum from HIV+ patients containing high levels of anti p17 antibodies was able to revert the down regulation of PPARγ and FXR in their monocytes [9].

Thereby, this implicates that the reset expressions of nuclear receptors, which are counter-inflammatory in nature, are likely instrumental in the progression of viral infection and the persistence of a chronic inflammatory state. Noteworthy, it has been demonstrated that members of nuclear receptor superfamily (i.e. GR, FXR, PXR, RAR and RXR) regulate immune responses and a proinflammatory state with the so called *transrepression pathway*, able to inhibit NF-KB [22]. It remains unclear whether the observed changes are causes or consequences of the characteristic inflammatory profile in HIV and whether NRs agonists can directly suppress residual inflammation.

In addition to their role in regulating immune responses, nuclear receptors regulate glucose and lipid metabolism [6]. Indeed, FXR has been demonstrated to regulate insulin secretion and sensitivity and FXR ligands reduce lipid and glucose levels in murine models of diabetes [23,24]. Similar beneficial effects on glucose metabolism have been shown for PXR [25], as well as for the fibrates (PPARα agonists) that are widely used for treatment of dyslipidemic conditions [26]. In short, abrogation of the expression of these genes might provide an explanation for immune dysfunction and dyslipidemia observed in HIV disease. Importantly, it has been demonstrated that FXR activation results in a slight reduction of HDL and in an induction of LDL [27], and therefore its abrogation might also provide some explanation for the hypo-cholesterolemia observed in HIV-positive persons.

Another important observation we made was that two nuclear receptors, VDR and PPARβ, appeared robustly induced in monocytes of HIV-infected patients. In addition, while the expressions of VDR and PPARβ normalize, the expression of LXR becomes significantly induced under HAART. This is an important observation, as a common side effect of LXR activation is hyper-triglyceridemia [28], and therefore its induction might provide a further explanation of the hyper-triglyceridemia observed in HAART.

In this study we have also seen that monocyte expression of CD36, whose transcription is primarily regulated by LXR, PPARγ and PXR, is markedly reduced by HIV infection. This suggests that the transcription of this gene is impaired in HIV monocytes. Noteworthy, we found the mRNA levels of CD36 significantly correlated with those of PPARγ in only HIV positive patients, thus providing a prognostic/diagnostic value to our profiles. Indeed, it has been previously demonstrated that HIV infection results in substantial decreases in serum total cholesterol, HDL and LDL levels and that subsequent HAART initiation is associated with increases in total cholesterol and LDL but little change in HDL [29]. Furthermore, patients with CD36 deficiency show increased levels of chylomicron remnants and small dense low-density lipoprotein (sdLDL) particles [30]. Thus, the reduction of CD36 gene expression we reported in this study might contribute to the high levels of total cholesterol in HIV positive persons taking HAART. These results also highlight a potential therapeutic role for PPARγ agonists in treating HIV associated dyslipidemia.

## Conclusions

Here, we have demonstrated that the transcriptome of several nuclear receptors is altered in monocytes during HIV infection. Furthermore, HAART failed to reverse this pattern with the exception for VDR and PPARβ. However, given that chronic inflammation and dyslipidemias are common in HIV-suppressed patients and that NRs seem to be directly or indirectly involved in this infection, our preliminary results suggest further investigations in a HIV positive population longitudinally followed from the starting of HAART and prompt to study whether nuclear receptor agonists can exercise any role in controlling residual inflammation and/or dyslipidemia.

## Abbreviations

FXR: Farnesoid X receptor; LXR: Liver X receptor; PXR: Pregnane X receptor; VDR: Vitamin D receptor; GR: Glucocorticoid receptor; RAR: Retinoic acid receptor; RXR: Retinoic X receptor; PPAR: Peroxisome proliferator activated receptor; HAART: Highly active antiretroviral therapy; MCP-1: Monocyte chemoattractant protein 1; ABCA-1: ATP-binding cassette sub-family a member 1; CD36: Cluster determinant 36 fatty acid translocase; ICAM-1: Intercellular adhesion molecule 1.

## Competing interest

The authors declare that they have no competing interests.

## Authors’ contributions

All the Authors have read and approved the final manuscript. BR, DF, SF and FB participated in the conception and design of the study, in the analysis and interpretation of data and in drafting the manuscript. AC, CD and ES carried out molecular biology experiments and statistical analysis of data.

## Pre-publication history

The pre-publication history for this paper can be accessed here:

http://www.biomedcentral.com/1471-2334/12/274/prepub
